# Work-Related Risk Factors by Severity for Acute Pesticide Poisoning Among Male Farmers in South Korea

**DOI:** 10.3390/ijerph10031100

**Published:** 2013-03-14

**Authors:** Ji-Hyun Kim, Jaeyoung Kim, Eun Shil Cha, Yousun Ko, Doo Hwan Kim, Won Jin Lee

**Affiliations:** 1 Department of Preventive Medicine, College of Medicine, Korea University, 126-1, 5 ga, Anam-dong, Seongbuk-gu, Seoul 136-705, Korea; E-Mails: muchic@korea.ac.kr (J.-H.K); eunshiri83@korea.ac.kr (E.S.C); junenine82@korea.ac.kr (Y.K.); 2 Department of Preventive Medicine, College of Medicine, Keimyung University, 1095, Dalgubeoldaero, Dalseo-gu, Daegu, Seoul 704-701, Korea; E-Mail: jaeykim@dsmc.or.kr; 3 Department of Sociology, Duksung Women’s University, 33, 144-Gil, Samyangro, Ssangmun-dong, Dobong-gu, Seoul 132-714, Korea; E-Mail: dhkim@ducksung.ac.kr

**Keywords:** agricultural workers, epidemiology, occupational exposure, poisoning, risk factors, severity

## Abstract

The objective of this study was to explore work-related risk factors of acute occupational pesticide poisoning among male farmers according to the severity of the poisoning. A nationwide sampling survey of male farmers was conducted in South Korea in 2011. A total of 1,958 male farmers were interviewed. Severity of occupational pesticide poisoning in 2010 was evaluated according to symptoms, types of treatment, and number of pesticide poisoning incidents per individual. A multinomial logistic regression model was used to estimate the odds ratio with 95% confidence intervals for risk factors of acute occupational pesticide poisoning. We found that the risk of acute occupational pesticide poisoning increased with lifetime days of pesticide application (OR = 1.74; 95% CI = 1.32–2.29), working a farm of three or more acres in size (OR = 1.49), not wearing personal protective equipment such as gloves (OR = 1.29) or masks (OR = 1.39). Those who engaged in inappropriate work behaviors such as not following pesticide label instructions (OR = 1.61), applying the pesticide in full sun (OR = 1.48), and applying the pesticide upwind (OR = 1.54) had a significantly increased risk of pesticide poisoning. There was no significant risk difference by type of farming. In addition, the magnitude of these risk factors did not differ significantly by severity of acute pesticide poisoning. In fact, our findings suggest that work-related risk factors contributed to the development of acute occupational pesticide poisoning without relation to its severity. Therefore, prevention strategies for reducing occupational pesticide poisoning, regardless of severity, should be recommended to all types of farming and the level of poisoning severity.

## 1. Introduction

Occupational pesticide poisoning is a major health problem among agricultural workers. It has been estimated that 1.3 billion workers are active in agricultural production worldwide [1]. Previous field studies from Nicaragua [2], Indonesia [3], Vietnam [4], Brazil [5], and China [6] have reported an occupational pesticide poisoning prevalence of 8.8% to 31%, based on self-reporting, although the study period and definition of poisoning varies between studies. In South Korea, a recent nationwide sampling survey conducted in 2011 demonstrated that the incidence rate of acute occupational pesticide poisoning was 24.7 per 100 male farmers, which corresponds to 209,512 cases in 2010 [7].

Although a number of studies have reported on risk factors for occupational pesticide poisoning ranging from demographics to work-related factors [6,8,9], there is a paucity of information in the available literature regarding difference, if any, in risk factors by severity of pesticide poisoning. However, identifying risk factors according to severity of poisoning is an important step in the preventing occupational pesticide poisoning and minimizing health effects through proper timely care. Most studies were also based on local surveys and little is known at the nationally representative level of the risk factors for occupational pesticide poisoning among farmers. The objective of this study, therefore, was to investigate risk factors for acute occupational pesticide poisoning according to its severity by using nationwide survey data for male farmers in South Korea.

## 2. Materials and Methods

### 2.1. Study Population and Method

The details of this study design and population have been described previously [7]. In brief, a nationwide sampling survey of male farmers in South Korea was conducted in 2011 using stratified multistage probability sampling methods and adopting in-person interviews with selected households from the 2010 Korean Agricultural Household registry data. A total of 1,958 households (98%) were interviewed among the 2,000 samples selected for the study.

Through a pilot study [10] and reference reviews, we selected 21 symptoms and signs for screening of acute pesticide poisoning in a field survey. These included nausea, vomiting, diarrhea, sore throat, runny nose, dyspnea, headache, dizziness, hyperactivity, profuse sweating, blurred vision, paresthesia, slurred speech, paralysis, chest pain, syncope, muscle weakness, skin irritation, eye irritation, lacrimation, and fatigue. Respondents were asked whether they had experienced any of the listed symptoms within 48 h of using pesticides during 2010. The questionnaire also included demographics and work-related factors such as age, level of education, marital status, annual income, smoking, alcohol drinking in past year, years applying pesticide, days applying pesticide, hours pesticide was applied, size of farm, type of farming, application method, use of personal protective equipment, and conformity to safety behaviors. Based on a pilot study [10], we selected six safety behaviors which were significantly related with acute pesticide poisoning. They include “did not follow label instructions”, “did not wear mask and gloves when mixing pesticides”, “applied pesticide in full sun which cause hot and humid temperatures to farmers”, “did not change clothes immediately after pesticide application”, “did not bathe with soap after pesticide application”, “applied pesticide upwind”. Lifetime pesticide application days was calculated based on multiplying the number of years of pesticide application by days of pesticide application (*i.e.*, years of use × days per year) and categorized into tertiles based on the number of participants.

### 2.2. Case Definition

Acute occupational pesticide poisoning was defined as a participant considering himself to have suffered any of the 21 listed symptoms or signs within 48 h of occupational pesticide use during 2010. Pesticide poisoning cases were classified by severity of symptoms or signs according to the proposal of Thundiyil *et al.* [11]. Severe cases were defined as any cases including symptoms of paralysis or syncope, while moderate cases were defined as suffering symptoms including vomiting, diarrhea, dyspnea, blurred vision, paresthesia, slurred speech, and chest pain. The remaining cases were classified as mild. Information regarding treatment for pesticide poisoning (*i.e.*, untreated, self-medicated, outpatient, and hospitalized) and number of episodes of pesticide poisoning was also collected in order to categorize the severity of pesticide poisoning.

### 2.3. Data Analysis

The distributions of major demographic characteristics and farming-related variables between poisoned and non-poisoned farmers were presented. Factors associated with pesticide poisoning were studied in a multinomial logistic regression model (SPSS, version 12.0.1). Odds ratios (ORs) and adjusted odds ratios with 95% confidence intervals (CIs) were derived from the models. The outcome variable was the severity of pesticide poisoning by symptom (*i.e.*, no poisoning, mild, moderate and severe), by type of care received (*i.e.*, no poisoning, untreated or self-treated, out- or inpatient), and by frequency of pesticide poisoning (*i.e.*, no poisoning, single poisoning, multiple poisoning). We evaluated possible confounders that may be associated with risk factors of acute pesticide poisoning. These included age, education, marital status, annual income, smoking, and alcohol drinking in the past year. Among those risk factors, age, income, and education level significantly changed the ORs by more than 10%. Due to multicollinearity of income and education level, however, the final models were adjusted for age and income. The non-pesticide poisoned group was used as a reference category. A statistically significant association between potential predictors of poisoning and outcome was declared when the *p*-value was less than 0.05.

### 2.4. Ethics Statement

This study was reviewed and approved by the Institutional Review Board of Korea University (KU-IRB-11–7-A-2). Informed written consent was voluntarily obtained from all individual study participants.

## 3. Results

### 3.1. Demographic Characteristics

Table 1 presents a comparison of demographics between poisoned and non-poisoned male farmers. A total of 449 farmers (22.9%) were identified as acute occupational pesticide poisoning cases. Age was inversely associated with the risk of pesticide poisoning, whereas annual income and education were positively associated with such risk. The other demographic factors surveyed showed no statistically significant association with acute occupational pesticide poisoning.

**Table 1:** Demographic characteristicsof male farmers surveyed nationwide by occupational pesticide poisoning in South Korea, 2010.

Characteristics *^a^*		No poisoning	Poisoning	Univariate OR (95% CI)
		Number (%)	Number (%)	
Total		1,509 (100)	449 (100)	-
Age (year)				
	<55	170 (11.3)	94 (20.9)	1.00
	55–59	112 (7.4)	50 (11.1)	0.81 (0.53 to 1.23)
	60–64	187 (12.4)	84 (18.7)	0.81 (0.57 to 1.17)
	65–69	224 (14.8)	60 (13.4)	0.48 (0.33 to 0.71)
	70–74	365 (24.2)	82 (18.3)	0.41 (0.29 to 0.58)
	≥75	451 (29.9)	79 (17.6)	0.32 (0.22 to 0.45)
Education				
	None	168 (11.2)	33 (7.4)	1.00
	Elementary school	671 (44.5)	174 (38.8)	1.32 (0.88 to 1.99)
	Middle school	305 (20.2)	96 (21.4)	1.60 (1.03 to 2.48)
	≥High school	364 (24.1)	145 (32.4)	2.03 (1.33 to 3.09)
Marital status				
	Married	1,390 (92.2)	418 (93.3)	1.00
	Never married	27 (1.8)	10 (2.2)	1.23 (0.59 to 2.57)
	Separated, divorced, widowed	90 (6.0)	20 (4.5)	0.74 (0.45 to 1.21)
Annual income (10,000 Korean won)^*b*^				
	<1,000	960 (63.8)	226 (50.5)	1.00
	1,000–1,999	273 (18.2)	81 (18.1)	1.26 (0.95 to 1.68)
	2,000–2,999	118 (7.8)	57 (12.8)	2.05 (1.45 to 2.91)
	≥3,000	153 (10.2)	83 (18.6)	2.30 (1.70 to 3.12)
Smoking status				
	Never	442 (29.3)	135 (30.1)	1.00
	Former	560 (37.2)	156 (34.7)	0.91 (0.70 to 1.19)
	Current	505 (33.5)	158 (35.2)	1.02 (0.79 to 1.33)
Number of alcoholic drinks over past year				
	None	284 (24.6)	86 (23.7)	1.00
	≤1 per week	260 (22.5)	93 (25.5)	1.18 (0.84 to 1.66)
	≥2 per week	610 (52.9)	185 (50.8)	1.00 (0.75 to 1.34)

*^a^* Numbers may not sum to total due to missing information; *^b^* 10,000 Korean won is equal to 9.31 US dollars as of February 2013.

### 3.2. Work-Related Factors

The comparison of work-related factors between poisoned and non-poisoned male farmers is presented in Table 2. Lifetime days of pesticide application significantly increased the risk of pesticide poisoning in a dose-dependent manner, with 1.74-fold increased risk in the highest category (95% CI = 1.32–2.29). 

**Table 2:** Work-related factors of male farmers surveyed nationwide by occupational pesticide poisoning in South Korea, 2010.

Characteristics *^a^*		No poisoning	Poisoning	Univariate OR (95% CI)	Adjusted OR *^b^* (95% CI)
		Number (%)	Number (%)		
Lifetime pesticide application days *^c^*					
	<90	496 (34.0)	124 (28.2)	1.00	1.00
	90–231	514 (35.3)	136 (30.9)	1.06 (0.81 to 1.39)	1.20 (0.90 to 1.59)
	≥232	448 (30.7)	180 (40.9)	1.61 (1.24 to 2.09)	1.74 (1.32 to 2.29)
Size of farm					
	<3 acres	989 (66.0)	225 (50.6)	1.00	1.00
	≥3 acres	509 (34.0)	220 (49.4)	1.90 (1.53 to 2.35)	1.49 (1.17 to 1.88)
Type of farming *^d^*					
	Rice	684 (45.4)	180 (40.0)	1.00	1.00
	Vegetable	163 (10.8)	36 (8.0)	0.84 (0.57 to 1.25)	0.81 (0.54 to 1.21)
	Greenhouse	35 (2.3)	7 (1.6)	0.76 (0.33 to 1.74)	0.55 (0.24 to 1.29)
	Fruit	74 (4.9)	34 (7.6)	1.75 (1.13 to 2.71)	1.35 (0.85 to 2.12)
	Mixed and other	553 (36.6)	192 (42.8)	1.32 (1.05 to 1.66)	1.14 (0.89 to 1.45)
Application method *^e^*					
	High pressure hand sprayer	975 (65.6)	301 (67.2)	1.00	1.00
	Manual backpack sprayer	170 (11.5)	37 (8.3)	0.71 (0.48 to 1.03)	0.74 (0.50 to 1.09)
	Speed sprayer (air blast)	38 (2.6)	19 (4.2)	1.62 (0.92 to 2.85)	1.32 (0.73 to 2.38)
	Mist or hand sprayer	47 (3.2)	5 (1.1)	0.35 (0.14 to 0.87)	0.43 (0.17 to 1.09)
	Air spray and other	253 (17.1)	86 (19.2)	1.10 (0.84 to 1.45)	1.06 (0.80 to1.40 )
Not wearing personal protective equipment *^f^*					
	Gloves	713 (48.2)	246 (54.9)	1.31 (1.06 to 1.62)	1.29 (1.04 to1.60)
	Safety glasses	1211(81.9)	377 (84.9)	1.24 (0.93 to 1.66)	1.30 (0.97 to1.76)
	Cap	247 (16.7)	77 (17.3)	1.04 (0.79 to 1.38)	0.89 (0.67 to1.20)
	Jacket	684 (46.3)	230 (51.3)	1.23 (0.99 to 1.51)	1.18 (0.95 to1.46)
	Pants	602 (40.7)	194 (43.4)	1.12 (0.90 to 1.38)	1.12 (0.90 to1.39)
	Boots	214 (14.5)	86 (19.2)	1.40 (1.06 to 1.84)	1.29 (0.97 to1.71)
	Masks	763 (51.6)	265 (59.0)	1.35 (1.09 to 1.68)	1.39 (1.11 to1.73)
Unsafe behaviors *^f^*					
	Did not follow label instructions	190 (12.9)	91 (20.3)	1.73 (1.31 to 2.28)	1.61 (1.21 to 2.13)
	Did not wear masks and gloves when mixing pesticides	603 (40.8)	209 (46.5)	1.26 (1.02 to 1.56)	1.24 (1.00 to 1.54)
	Applied pesticides in full sun	167 (11.3)	74 (16.5)	1.55 (1.15 to 2.08)	1.48 (1.09 to 2.01)
	Did not change cloths immediately after pesticide application	86 (5.8)	23 (5.1)	0.87 (0.55 to 1.40)	0.90 (0.55 to1.46 )
	Did not bathe with soap after pesticide application	99 (6.7)	28 (6.2)	0.93 (0.60 to 1.43)	1.00 (0.64 to1.56 )
	Applied pesticides upwind	289 (19.6)	130 (29.0)	1.67 (1.31 to 2.13)	1.54 (1.20 to1.97 )

*^a^* Numbers may not sum to total due to missing information; *^b^* Adjusted for age and income; *^c^* Lifetime pesticide application days was calculated based on multiplying the number of years of pesticide application by days of pesticide application (*i.e.*, years of use x days per year) and categorized into tertile based on the number of participants; *^d^* Reference was the respondents who did not engage in the corresponding type of farming; *^e^* Reference was the respondents who did not use the corresponding methods; *^f^* Reference was the respondents who used corresponding personal protective equipment or applied safety behaviors.

Working on a farm of three or more acres in size, not wearing gloves and masks, and engaging in inappropriate behaviors at work such as did not follow label instructions, did not wear mask and gloves when mixing pesticides, pesticide application in full sun, and applying pesticide upwind, were all significantly associated with an increased risk of occupational pesticide poisoning.

**Table 3:** ORs and 95% CIs for pesticide poisoning by symptom severity and work-related factors among South Korean male farmers, 2010.

Characteristics *^a^*		No poisoning	Mild		Moderate/Severe	
		Number	Number	Adjusted OR *^b^* (95% CI)	Number	Adjusted OR *^b^* (95% CI)
Lifetime pesticide application days *^c^*						
	<90	496	83	1.00	41	1.00
	90–231	514	95	1.28 (0.92 to 1.78)	41	1.14 (0.72 to 1.81)
	≥232	448	108	1.56 (1.13 to 2.17)	72	2.36 (1.55 to 3.60)
Size of farm						
	<3 acres	989	151	1.00	74	1.00
	≥3 acres	509	139	1.37 (1.04 to 1.80)	81	1.75 (1.22 to 2.50)
Type of farming *^d^*						
	Rice	684	120	1.00	60	1.00
	Vegetable	163	27	0.91 (0.58 to 1.44)	9	0.59 (0.29 to 1.22)
	Greenhouse	35	5	0.61 (0.23 to 1.60)	2	0.50 (0.12 to 2.14)
	Fruit	74	25	1.46 (0.88 to 2.44)	9	1.13 (0.53 to 2.41)
	Mixed and other	553	116	1.04 (0.78 to 1.38)	76	1.39 (0.97 to 2.00)
Application method *^e^*						
	High pressure hand sprayer	975	206	1.00	95	1.00
	Manual backpack sprayer	170	21	0.61 (0.38 to 0.99)	16	1.02 (0.58 to 1.78)
	Speed sprayer (air blast)	38	12	1.12 (0.56 to 2.24)	7	1.69 (0.72 to 3.99)
	Mist or hand sprayer	47	4	0.50 (0.18 to 1.40)	1	0.27 (0.04 to 1.97)
	Air spray and other	253	49	0.87 (0.62 to 1.23)	37	1.47 (0.98 to 2.21)
Not wearing personal protective equipment *^f^*						
	Gloves	713	166	1.38 (1.07 to 1.79)	80	1.12 (0.80 to 1.56)
	Safety glasses	1211	244	1.27 (0.89 to 1.80)	133	1.41 (0.87 to 2.26)
	Cap	247	49	0.86 (0.61 to 1.22)	28	0.89 (0.57 to 1.39)
	Jacket	684	146	1.10 (0.85 to 1.42)	84	1.29 (0.92 to 1.81)
	Pants	602	116	0.96 (0.74 to 1.25)	78	1.42 (1.01 to 1.98)
	Boots	214	48	1.04 (0.74 to 1.48)	38	1.76 (1.18 to 2.62)
	Masks	763	170	1.33 (1.03 to 1.72)	95	1.46 (1.04 to 2.06)
Unsafe behaviors *^f^*						
	Did not follow label instructions	190	58	1.58 (1.13 to 2.20)	33	1.61 (1.05 to 2.46)
	Did not wear masks and gloves when mixing pesticides	603	142	1.35 (1.04 to 1.74)	67	1.05 (0.75 to 1.47)
	Applied pesticides in full sun	167	48	1.45 (1.02 to 2.07)	26	1.43 (0.90 to 2.27)
	Did not change cloths immediately after pesticide application	86	13	0.73 (0.40 to 1.33)	10	1.09 (0.55 to 2.17)
	Did not bathe with soap after pesticide application	99	18	0.93 (0.55 to 1.58)	10	0.98 (0.50 to 1.94)
	Applied pesticides upwind	289	79	1.37 (1.02 to 1.84)	51	1.81 (1.25 to 2.61)

*^a^* Numbers may not sum to total due to missing information; *^b^* Adjusted for age and income; *^c^* Lifetime pesticide application days calculated based on multiplying the number of years of pesticide application by days of pesticide application (*i.e.*, years of use x days per year) and categorized into tertile based on the number of participants; *^d^* Reference was the respondents who did not engage in the corresponding type of farming; *^e^* Reference was the respondents who did not use the corresponding methods; *^f^* Reference was the respondents who used corresponding personal protective equipment or applied safety behaviors.

### 3.3. Symptom Severity

According to severity of symptoms, lifetime days of pesticide application, working a farm of three or more acres in size, not wearing personal protective equipment such as pants, boots, masks, and inappropriate safety behaviors such as did not follow label instructions, and applying pesticide upwind were significantly associated with both mild and moderate/severe poisoning groups (Table 3). Types of farming and application method were not significantly related with the risk of occupational pesticide poisoning. The strength of association of work-related risk factors such as lifetime days of pesticide application, size of farm, and unsafe behaviors were generally similar between groups. 

### 3.4. Type of Care Received

According to type of care received, pesticide application days, farm size, and the application of pesticide with a speed sprayer tended to be more significantly correlated with the hospital-treated group than with those in the untreated/self-medicated group, whereas not wearing personal protective equipment and inappropriate behaviors showed an opposite trend (Table 4). However, the magnitudes of risk by type of care received were not significantly different between groups. 

**Table 4:** ORs and 95% CIs for pesticide poisoning by type of care received and work-related factors among South Korean male farmers, 2010.

Characteristics *^a^*		No poisoning	Untreated/Self-medicated		Outpatient/Inpatient	
		Number	Number	Adjusted OR *^b^* (95% CI)	Number	Adjusted OR *^b^* (95% CI)
Lifetime pesticide application days *^c^*						
	<90	496	111	1.00	11	1.00
	90–231	514	115	1.18 (0.87 to 1.59)	18	1.72 (0.80 to 3.71)
	≥232	448	153	1.73 (1.30 to 2.32)	24	2.71 (1.29 to 5.68)
Size of farm						
	< 3acres	989	194	1.00	26	1.00
	≥ 3acres	509	190	1.43 (1.12 to1.84)	27	2.10 (1.17 to 3.78)
Type of farming *^d^*						
	Rice	684	161	1.00	17	1.00
	Vegetable	163	31	0.77 (0.50 to 1.18)	3	0.72 (0.21 to 2.49)
	Greenhouse	35	5	0.44 (0.17 to 1.15)	2	2.19 (0.48 to 9.97)
	Fruit	74	30	1.29 (0.80 to 2.07)	4	2.16 (0.70 to 6.68)
	Mixed and other	553	161	1.06 (0.83 to 1.37)	27	1.95 (1.04 to 3.63)
Application method *^e^*						
	High pressure hand sprayer	975	266	1.00	31	1.00
	Manual backpack sprayer	170	31	0.70 (0.47 to 1.06)	4	0.75 (0.26 to 2.15)
	Speed sprayer (air blast)	38	15	1.09 (0.58 to 2.06)	4	3.64 (1.18 to 11.24)
	Mist or hand sprayer	47	4	0.39 (0.14 to 1.10)	0	-
	Air spray and other	253	71	0.98 (0.72 to 1.32)	14	1.75 (0.92 to 3.35)
Not wearing personal protective equipment *^f^*						
	Gloves	713	216	1.33 (1.05 to 1.67)	24	0.89 (0.51 to 1.54)
	Safety glasses	1211	330	1.41 (1.02 to 1.94)	41	0.84 (0.43 to 1.66)
	Cap	247	67	0.86 (0.63 to 1.17)	9	0.99 (0.47 to 2.07)
	Jacket	684	199	1.16 (0.92 to 1.46)	26	1.10 (0.64 to 1.91)
	Pants	602	167	1.09 (0.87 to 1.38)	23	1.11 (0.64 to 1.93)
	Boots	214	72	1.21 (0.89 to 1.63)	11	1.52 (0.77 to 3.00)
	Masks	763	231	1.40 (1.11 to 1.77)	28	1.06 (0.61 to 1.83)
Unsafe behaviors *^f^*						
	Did not follow label instructions	190	80	1.61 (1.20 to 2.17)	8	1.16 (0.54 to 2.50)
	Did not wear masks and gloves when mixing pesticides	603	182	1.25 (0.99 to 1.57)	21	0.94 (0.54 to 1.65)
	Applied pesticides in full sun	167	65	1.46 (1.06 to 2.01)	7	1.18 (0.53 to 2.67)
	Did not change cloths immediately after pesticide application	86	21	0.90 (0.54 to 1.50)	1	0.32 (0.04 to 2.32)
	Did not bathe with soap after pesticide application	99	25	0.99 (0.62 to 1.58)	2	0.56 (0.13 to 2.33)
	Applied pesticides upwind	289	112	1.49 (1.14 to 1.94)	13	1.32 (0.69 to 2.51)

*^a^* Numbers may not sum to total due to missing information; *^b^* Adjusted for age and income; *^c^* Lifetime pesticide application days calculated based on multiplying the number of years of pesticide application by days of pesticide application (*i.e.*, years of use x days per year) and categorized into tertile based on the number of participants; *^d^* Reference was the respondents who did not engage in the corresponding type of farming; *^e^* Reference was the respondents who did not use the corresponding methods; *^f^* Reference was the respondents who used corresponding personal protective equipment or applied safety behaviors.

### 3.5. Number of Occupational Pesticide Poisonings

Those with multiple poisonings showed more significant associations with pesticide application days, farm size, not wearing gloves or masks, and inappropriate safety behaviors such as not following instructions and dosage according to guidelines, applying the pesticide in full sun, and applying the pesticide upwind than did those in the single poisoning group (Table 5). However, the risk for occupational pesticide poisoning by work-related factors was not materially different between both single- and multiple-poisoning groups.

**Table 5:** ORs and 95% CIs for pesticide poisoning by number of poisonings and work-related factors among South Korean male farmers, 2010.

Characteristics *^a^*		No poisoning	Single poisoning		Multiple poisoning	
		Number	Number	Adjusted OR *^b^* (95% CI)	Number	Adjusted OR *^b^* (95% CI)
Lifetime pesticide application days *^c^*						
	<90	496	55	1.00	65	1.00
	90–231	514	32	0.63 (0.40 to 1.01)	103	1.83 (1.29 to 2.58)
	≥232	448	39	0.81 (0.52 to 1.26)	133	2.69 (1.92 to 3.77)
Size of farm						
	<3 acres	989	68	1.00	152	1.00
	≥3 acres	509	60	1.32 (0.89 to1.96)	152	1.54 (1.18 to 2.02)
Type of farming *^d^*						
	Rice	684	60	1.00	118	1.00
	Vegetable	163	10	0.67 (0.34 to 1.35)	25	0.84 (0.53 to 1.35)
	Greenhouse	35	3	0.73 (0.21 to 2.47)	3	0.38 (0.11 to 1.28)
	Fruit	74	8	0.92 (0.41 to 2.03)	26	1.62 (0.98 to 2.68)
	Mixed and other	553	48	0.84 (0.56 to 1.27)	135	1.25 (0.95 to 1.66)
Application method *^e^*						
	High pressure hand sprayer	975	84	1.00	212	1.00
	Manual backpack sprayer	170	13	0.94 (0.51 to 1.72)	23	0.65 (0.41 to 1.04)
	Speed sprayer (air blast)	38	6	1.41 (0.56 to 3.51)	12	1.20 (0.60 to 2.38)
	Mist or hand sprayer	47	3	0.92 (0.28 to 3.04)	2	0.24 (0.06 to 1.00)
	Air spray and other	253	22	0.97 (0.59 to 1.59)	58	1.02 (0.73 to 1.41)
Not wearing personal protective equipment *^f^*						
	Gloves	713	72	1.37 (0.94 to 1.97)	172	1.35 (1.05 to 1.73)
	Safety glasses	1211	109	1.34 (0.80 to 2.23)	258	1.34 (0.95 to 1.90)
	Cap	247	29	1.28 (0.82 to 1.99)	45	0.71 (0.50 to 1.02)
	Jacket	684	69	1.29 (0.89 to 1.85)	156	1.14 (0.89 to 1.46)
	Pants	602	60	1.30 (0.90 to 1.87)	128	1.03 (0.80 to 1.33)
	Boots	214	27	1.42 (0.90 to 2.24)	56	1.20 (0.86 to 1.67)
	Masks	763	70	1.15 (0.80 to 1.66)	187	1.48 (1.15 to 1.91)
Unsafe behaviors *^f^*						
	Did not follow label instructions	190	23	1.41 (0.87 to 2.28)	67	1.73 (1.26 to 2.38)
	Did not wear masks and gloves when mixing pesticides	603	59	1.21 (0.84 to 1.74)	146	1.27 (0.99 to 1.64)
	Applied pesticides in full sun	167	13	0.83 (0.46 to1.52)	59	1.73 (1.24 to 2.42)
	Did not change cloths immediately after pesticide application	86	2	0.25 (0.06 to1.02)	21	1.16 (0.70 to 1.92)
	Did not bathe with soap after pesticide application	99	3	0.34 (0.11 to1.09)	24	1.21 (0.75 to 1.94)
	Applied pesticides upwind	289	32	1.23 (0.80 to1.88)	93	1.61 (1.22 to 2.14)

*^a^* Numbers may not sum to total due to missing information; *^b^* Adjusted for age and income; *^c^* Lifetime pesticide application days calculated based on multiplying the number of years of pesticide application by days of pesticide application (*i.e.*, years of use x days per year) and categorized into tertile based on the number of participants; *^d^* Reference was the respondents who did not engage in the corresponding type of farming; *^e^* Reference was the respondents who did not use the corresponding methods; *^f^* Reference was the respondents who used corresponding personal protective equipment or applied safety behaviors.

## 4. Discussion

This study demonstrates through a nationwide sampling survey among male farmers that lifetime pesticide application days, farm size, not wearing gloves or mask, and inappropriate safety behaviors such as “did not follow label instructions”, “pesticide application in full sun”, and “applying pesticide upwind” are significant risk factors for acute occupational pesticide poisoning. These factors did not show any significant difference by pesticide poisoning severity, which suggests that they may not contribute to the development of pesticide poisoning in a dose-dependent manner. Our results emphasize that prevention strategies for minimizing occupational pesticide poisoning should be recommended across all types of severity for occupational pesticide poisoning, not simply focusing on severe poisoning. The primary targets for minimizing occupational pesticide poisoning would be reducing the number of pesticide application, wearing personal protective equipment, and following recommended safety behaviors, which are shown here to be strong risk factors across all severities of cases.

Previously, specific types of farmers, such as orchardists [12] and greenhouse farmers [13] were reported to have an increased risk of pesticide poisoning in South Korea. However, no significant difference was found by type of farming in this study. Although the risk of pesticide poisoning was non-significantly increased among hospital visits cases compared to untreated/self-medicated cases. Since there are large variations in work practices within specific farm types, direct work-related factors such as personal protective equipment and safety behaviors may contribute to occupational pesticide poisoning to a greater degree than type of farming. Therefore, we suggest that it is important to apply prevention strategies to all farmers rather than simply focusing on specific type of farmers.

Consistent with previous studies [8,14,15], our risk for pesticide poisoning increased with increasing time of pesticide application, although the risk did vary according to work-related factors. There is no doubt that reducing the number of pesticide application is the one of most important risk factors for the prevention of occupational pesticide poisoning. Therefore, developing new farming methods for reducing the overall number of pesticide applications should be encouraged in this regard. The increased risk of pesticide poisoning by farm size was also likely due to longer application times and more frequent pesticide application, which may increase the possibility of pesticide poisoning [16,17].

Our study showed an increased risk of pesticide poisoning with the lack of proper use of gloves or masks as personal protective equipment during pesticide application, as did previous studies [8,18]. The hands have been reported to be the body part most exposed to pesticides in most circumstances [19,20] and using gloves has been reported to significantly reduce pesticide exposure [21,22]. Respiratory protection is also important for lowering exposure to pesticides, especially for highly volatile pesticides [23]. Therefore, we confirmed that supplying personal protective equipment, especially gloves and masks, is important to the prevention of occupational pesticide poisoning among male farmers in South Korea.

Among safety behaviors, failing to follow label instructions, pesticide application in full sun, and applying the pesticide upwind significantly increased risk, which was similar with results from other studies [24,25]. This may due to elevated temperatures discouraging the use of personal protective equipment [26]. Dermal exposure may also increase markedly when pesticides were applied upwind. Regulatory control and educational efforts related to these activities should be addressed in order to reduce occupational pesticide poisoning.

Among hospital visits cases, pesticide poisoning was significantly increased by using a speed-sprayer, although severe-symptom cases did not show similar results. Previous studies have reported that pesticide poisoning has frequently occurred among pesticide applicators using a speed sprayer type compared with other application methods [23,27]. This is due to speed sprays being a powerful and high-velocity method, applied in an upward direction, so the applicator experiences a greater chance to be exposed by inhalation or dermal than with other types of application.

Similar to previous studies [8,28], our results demonstrated that the risk for occupational pesticide poisoning decreased with increasing age, which suggests that relatively younger farmers engage in more outdoor work than do older farmers. Our result on the increased risk among pesticide applicators with the increase in income may imply large farm size, which would consequently require more frequent or larger amounts of pesticide application. Pesticide poisoning was significantly increased with the increase in education level in this study, which was consistent with other research [6,8]. However, education level may impact the use of personal protective equipment differently by nation.

This study includes some limitations. First, minor cases may be over-reported because symptoms related to pesticide poisoning could also have been attributed to other work-related or pre-existing medication conditions. This would dilute the true associations found. However, severe cases would have been reported more correctly than milder cases since recall bias may not significantly affect severe injuries [29]. Second, self-reported data on pesticide use and work-related risk factors may introduce potential bias. However, the questionnaire-based survey method which we applied is commonly used in public health since information related with risk factors or pesticide poisoning, such as frequency, duration, or type of application, can be obtained by this form of questioning. Third, this study lacks detailed information on risk factors for pesticide poisoning such as socio-cultural or behavioral factors, which should be investigated in a further study.

Despite these limitations, we found that the contribution of work-related risk factors for occupational pesticide poisoning did not differ by severity or number of pesticide poisonings. This is a nationwide representative survey among male farmers and features information allowing the categorization of pesticide poisoning, such as symptom severity, type of treatment received, and number of poisonings. These findings suggested that preventive strategies for occupational pesticide poisoning in South Korea should take the approach of reducing overall work-related risk factors and address all types of farmers or poisoning cases rather than specific types.

## References

[1] ILO The ILO Programme on Occupational Safety and Health in Agriculture. http://www.ilo.org/safework/areasofwork/WCMS_117367/lang--en/index.htm.

[2] Keifer M., Rivas F., Moon J.D., Checkoway H. (1996). Symptoms and cholinesterase activity among rural residents living near cotton fields in nicaragua. Occup. Environ. Med..

[3] Kishi M., Hirschhorn N., Djajadisastra M., Satterlee L.N., Strowman S., Dilts R. (1995). Relationship of pesticide spraying to signs and symptoms in Indonesian farmers. Scand. J. Work Environ. Health.

[4] Murphy H.H., Hoan N.P., Matteson P., Abubakar A.L. (2002). Farmers’ self-surveillance of pesticide poisoning: A 12-month pilot in northern Vietnam. Int. J. Occup. Environ. Health.

[5] Faria N.M., Rosa J.A., Facchini L.A. (2009). Poisoning by pesticides among family fruit farmers, Bento Goncalves, Southern Brazil. Rev. Saude Publica.

[6] Zhang X., Zhao W., Jing R., Wheeler K., Smith G.A., Stallones L., Xiang H. (2011). Work-related pesticide poisoning among farmers in two villages of Southern China: A cross-sectional survey. BMC Public Health.

[7] Lee W.J., Cha E.S., Park J., Ko Y., Kim H.J., Kim J. (2012). Incidence of acute occupational pesticide poisoning among male farmers in South Korea. Am. J. Ind. Med..

[8] Bell E.M., Sandler D.P., Alavanja M.C. (2006). High pesticide exposure events among farmers and spouses enrolled in the Agricultural Health Study. J. Agric. Saf. Health.

[9] Solomon C., Poole J., Palmer K.T., Peveler R., Coggon D. (2007). Acute symptoms following work with pesticides. Occup. Med. (Lond).

[10] Kim H.J., Cha E.S., Moon E.K., Ko Y., Kim J., Jeong M., Lee W.J. (2011). A pilot study for pesticide poisoning symptoms and information on pesticide use among farmers. J. Environ. Health Sci..

[11] Thundiyil J.G., Stober J., Besbelli N., Pronczuk J. (2008). Acute pesticide poisoning: A proposed classification tool. Bull. World Health Organ..

[12] Kwon Y., Kang T., Kim K., Lee K., Ju Y., Song J. (2004). The relationship between pesticide exposure and central nervous system symptoms. Korean J. Rural Med..

[13] Lee J.Y., Park J.H., Kim D. (1994). A survey on physical complaints related with farmers’ syndrome of vinylhouse and non-vinylhouse farmers. Korean J. Prev. Med..

[14] Jors E., Morant R.C., Aguilar G.C., Huici O., Lander F., Baelum J., Konradsen F. (2006). Occupational pesticide intoxications among farmers in bolivia: A cross-sectional study. Environ. Health.

[15] Jensen H.K., Konradsen F., Jors E., Petersen J.H., Dalsgaard A. (2011). Pesticide use and self-reported symptoms of acute pesticide poisoning among aquatic farmers in Phnom Penh, Cambodia. J. Toxicol..

[16] Alavanja M.C., Sandler D.P., McDonnell C.J., Lynch C.F., Pennybacker M., Zahm S.H., Mage D.T., Steen W.C., Wintersteen W., Blair A. (1999). Characteristics of pesticide use in a pesticide applicator cohort: The Agricultural Health Study. Environ. Res..

[17] Lee K.M., Min S.Y., Chun M.H. (2000). A study on the health effects of pesticide exposure among farmers. Korean J. Rural Med..

[18] Taneepanichskul N., Norkaew S., Siriwong W., Robson M.G. (2012). Health effects related to pesticide using and practicing among chilli-growing farmers, northeastern, Thailand. J. Med. Med. Sci..

[19] van der Jagt K., Tielemans E., Links I., Brouwer D., van Hemmen J. (2004). Effectiveness of personal protective equipment: Relevance of dermal and inhalation exposure to chlorpyrifos among pest control operators. J. Occup. Environ. Hyg..

[20] Baldi I., Lebailly P., Jean S., Rougetet L., Dulaurent S., Marquet P. (2006). Pesticide contamination of workers in vineyards in France. J. Expo. Sci. Environ. Epidemiol..

[21] Krieger R.I., Dinoff T.M. (2000). Captan fungicide exposures of strawberry harvesters using thpi as a urinary biomarker. Arch. Environ. Contam. Toxicol..

[22] Hines C.J., Deddens J.A., Coble J., Kamel F., Alavanja M.C. (2011). Determinants of captan air and dermal exposures among orchard pesticide applicators in the agricultural health study. Ann. Occup. Hyg..

[23] Dowling K.C., Seiber J.N. (2002). Importance of respiratory exposure to pesticides among agricultural populations. Int. J. Toxicol..

[24] Shin D.C., Kim H.J., Jung S.H., Park C.Y., Lee S.Y., Kim C.B. (1998). Pesticide poisoning and its related factors among Korean farmers. Med. Lav..

[25] Mekonnen Y., Agonafir T. (2002). Pesticide sprayers’ knowledge, attitude and practice of pesticide use on agricultural farms of Ethiopia. Occup. Med. (Lond).

[26] Park E.K., Hannaford-Turner K., Lee H.J. (2009). Use of personal protective equipment in agricultural workers under hot and humid conditions. Ind. Health.

[27] Hong S.S., Jeong M., Park K.H., You A.S., Park Y.K., Lee J.B., Kim C.S., Shin J.S., Park J.E. (2010). The preliminary operator risk assessment of high toxicological pesticides in Korea. Korean J. Pestic. Sci..

[28] Corriols M. (2009). Acute Pesticide Poisonings in Nicaragua: Underreporting, Incidence and Determinants. Ph.D Thesis.

[29] Moshiro C., Heuch I., Astrom A.N., Setel P., Kvale G. (2005). Effect of recall on estimation of non-fatal injury rates: A community based study in Tanzania. Inj. Prev..

